# HRV-Based Respiratory-Rate Estimation in Older Adults: Error Modeling and Pilot Validation for Wearable Sleep Monitoring

**DOI:** 10.3390/clockssleep8030054

**Published:** 2026-08-31

**Authors:** Emi Yuda, Naoya Morikawa, Junichiro Hayano

**Affiliations:** 1 Innovation Center for Semiconductor and Digital Future, Mie University, Tsu 514-8507, Japan; 2 Department of Management Science and Technology, Graduate School of Engineering, Tohoku University, Sendai 980-8579, Japan; 3 Department of Information Engineering, Graduate School of Engineering, Mie University, Tsu 514-8507, Japan; 426m532@m.mie-u.ac.jp; 4 School of Medicine, Nagoya City University, Nagoya 467-8601, Japan; hayano@acm.org

**Keywords:** heart-rate variability, respiratory sinus arrhythmia, respiratory-rate estimation, wearable sensors, sleep monitoring, Welch spectral analysis, HF component, signal quality

## Abstract

Respiratory frequency is a critical biomarker in sleep medicine and circadian biology. We investigated whether the high-frequency (HF) component of heart-rate variability (HRV)—which reflects respiratory sinus arrhythmia (RSA)—can serve as a non-invasive proxy for breathing rate estimation from ECG or PPG. We employed a two-stage validation design: (1) a physiologically calibrated simulation study (N = 30 per condition, five conditions, 180 s recordings) for controlled error characterization using the PhysioNet ECG-ID Database (Electrocardiogram Identification Database, DOI: 10.13026/C2XW26) as the processing pipeline reference; and (2) pilot real-data validation in N = 10 older adult participants (mean age 71.3 years) with simultaneous ECG and thermistor respiratory reference measured using the East Medic Biotope Mini (1000 Hz). Results: Under controlled resting conditions, simulation yielded MAE = 0.46 bpm (SNR = 4.8). An empirical error formula MAE = 2.017 × SNR^(−1.187) (R^2^ = 0.71) was derived. In the real-data validation, 4/10 participants achieved MAE ≤ 2.0 bpm; the remaining 6/10 showed errors of 6–17 bpm attributable to non-respiratory HF oscillations, harmonic confusion, and breathing rate variability. The HF-peak method is reliable when SNR is high and breathing is regular but requires additional quality criteria beyond SNR alone in older adult populations where non-respiratory HF oscillations may confound spectral peak detection.

## 1. Introduction

Respiratory frequency is one of the four primary vital signs and a fundamental biomarker in sleep medicine, autonomic neuroscience, and circadian biology [1,2]. Breathing transitions from cortical to autonomous brainstem control during sleep, producing stage-dependent patterns that index homeostatic state, autonomic balance, and sleep quality [3]. Continuous, non-invasive respiratory monitoring is therefore central to both clinical polysomnography and emerging wearable circadian research. The high-frequency (HF) component of HRV (0.15–0.40 Hz) arises from respiratory sinus arrhythmia (RSA)—the vagally mediated beat-to-beat modulation of heart rate by breathing [4]. Because this coupling encodes the breathing cycle directly in the RR interval series, the HF spectral peak frequency provides a non-invasive estimate of respiratory frequency from any device that detects cardiac cycles [5,6]. This principle underlies respiratory-rate estimation in commercial wearables (Apple Watch, Polar, Garmin, WHOOP) [7,8].

The primary respiratory frequency estimate from HRV is:

(1) $$f_{resp}[Hz]=argmax\_\{f\;in\;[0.15,0.40]\}{PSD}_{HF}(f)$$

yielding breathing rate BR [bpm] = 60 × f_resp_. Despite widespread wearable deployment, no published study has provided: (1) a parametric error prediction formula based on spectral signal quality; (2) systematic validation across clinically relevant signal quality conditions; or (3) real-data validation in older adult populations where RSA coupling may be weakened and non-respiratory HF oscillations may confound spectral peak detection. We address these gaps through a two-stage approach. First, we conduct a simulation study using a physiologically calibrated RR-interval model across five signal quality conditions, deriving an empirical error formula based on HF signal-to-noise ratio (SNR). Second, we validate the method against thermistor-based respiratory ground truth in N = 10 older adult community-dwelling volunteers. Together, these components provide a principled characterization of the method’s accuracy, quality predictors, and failure modes across the population most relevant.

## 2. Results

### 2.1. Simulation: Condition-Wise Accuracy

**Figure 1:**
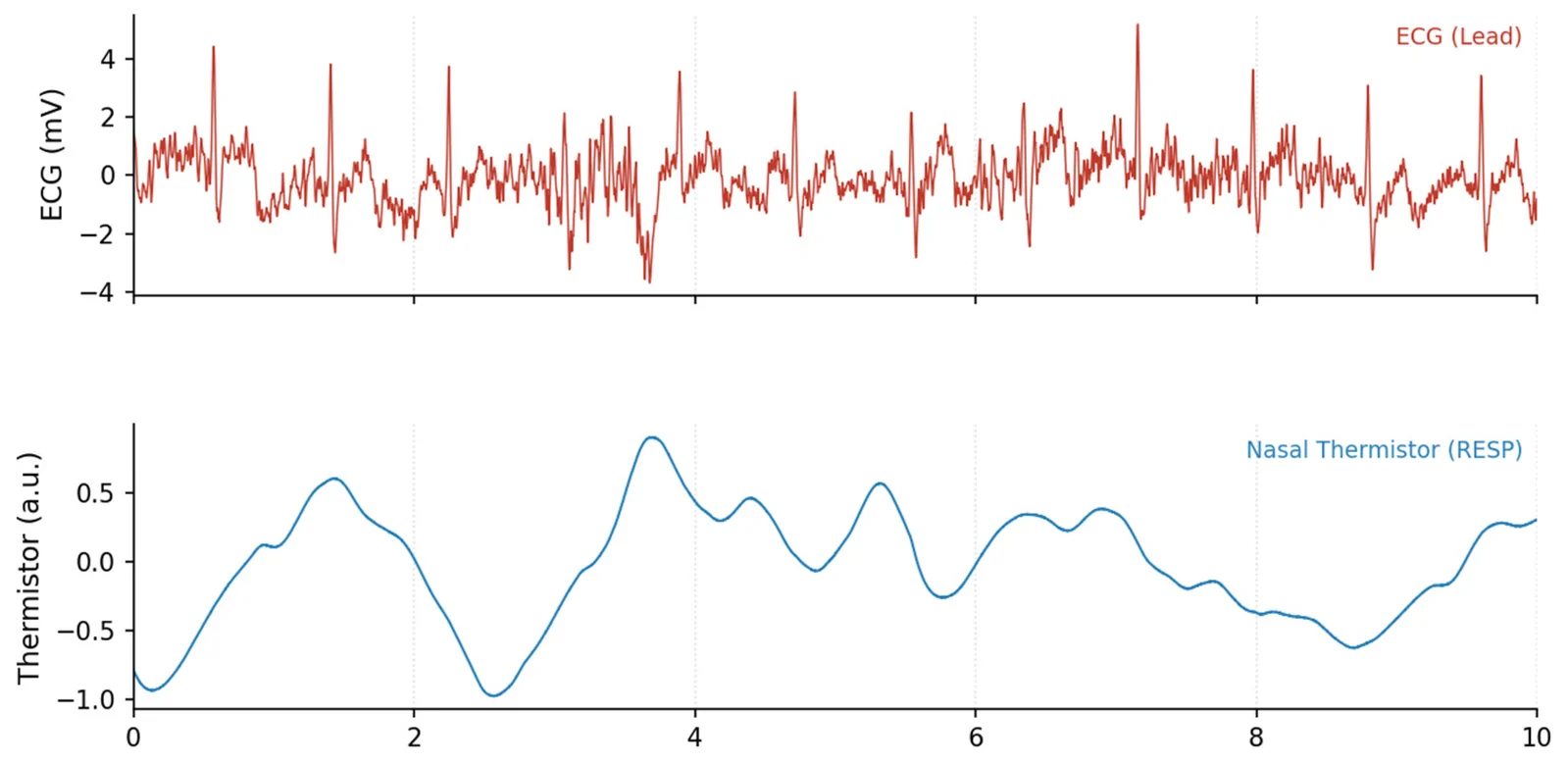
Ten-second ECG and respiratory waveform from a single participant (74-year-old male).

Figure 1 illustrates a representative 10 s segment of ECG (red) and thermistor-derived respiration (blue) from a single participant (74-year-old male), with the horizontal axis indicating time in seconds. This example highlights the characteristic coupling between respiratory airflow and RSA-driven heart-rate modulation, forming the basis for the simulation framework.

Table 1 shows respiratory-rate estimation accuracy across the five simulated signal quality conditions. Under normal resting conditions, estimation was highly accurate (MAE = 0.46 bpm, SNR = 4.8). Respiratory irregularity was the dominant error source (mild: MAE = 3.95 bpm; severe: MAE = 3.51 bpm), confirming that peak-frequency instability—rather than RSA amplitude per se—drives estimation error. RSA amplitude reduction alone caused minimal frequency-estimation error (MAE = 0.51 bpm, SNR = 4.0), challenging the commonly cited assumption [5,6,9] that RSA attenuation directly causes 2–4 bpm error.

The red trace shows the ECG signal, and the blue trace shows the thermistor-derived respiratory waveform. The horizontal axis indicates time in seconds. ECG was recorded simultaneously with thermistor-based respiration. The respiratory signal reflects airflow-related temperature changes measured at the nostrils. The time axis is expressed in seconds.

### 2.2. Empirical Error Estimation Formula

Log-linear regression of MAE on HF-SNR across 600 synthetic recordings yielded (R^2^ = 0.71 on log–log scale; the residual 29% of variance reflects non-linear factor interactions not captured by the univariate model):

(2) $$MAE=2.017\cdot {SNR}^{-1.187}$$

An SNR threshold of 2.0 corresponds to predicted MAE = 0.89 bpm, providing a principled real-time quality-gating criterion without requiring a respiratory reference signal. The formula is intended as a practical quality-gating tool; recalibration against PSG respiratory ground truth is recommended before clinical deployment (Table 2).

We summarized the predicted respiratory-rate estimation error (MAE) as a function of HF-SNR based on Equation (5). The empirical model provides a monotonic relationship in which higher HF-SNR yields lower estimation error. This allows HF-SNR to be interpreted as a direct indicator of signal quality for respiratory-rate extraction. At low HF-SNR values (1.2–1.5), the predicted MAE exceeds 1 bpm, corresponding to marginal or low-quality conditions. At HF-SNR = 2.0, the MAE falls below 1 bpm (0.89 bpm), representing a moderate quality level and defining a practical quality-gate threshold for reliable estimation. Further increases in HF-SNR (3.0–8.0) reduce MAE to below 0.6 bpm, corresponding to good, high, or excellent signal quality. Overall, Table 3 provides a quantitative mapping from HF-SNR to expected estimation accuracy, enabling real-time assessment of whether a given recording meets the quality requirements for robust respiratory-rate estimation.

### 2.3. RSA Attenuation Does Not Shift HF Peak Frequency

Critically, RSA amplitude reduction alone (condition 3: A_RSA = 0.02 s vs. 0.05 s in normal rest) produced MAE = 0.51 bpm—statistically indistinguishable from normal rest (MAE = 0.46 bpm). RSA attenuation reduces HF power but does not shift the peak frequency as long as SNR remains above approximately 1.5. Only when RSA attenuation is concurrent with irregularity or noise—thereby degrading SNR—does estimation error increase appreciably. This corrects the commonly cited figure of “2–4 bpm error from RSA attenuation,” which conflates amplitude reduction with frequency estimation error.

### 2.4. Real-Data Validation: 10 Elderly Participants

Table 2 shows per-subject results from the 10-subject real-data validation cohort (mean age 71.3 ± 6.1 years; five male, five female; measurement dates 1–2 March 2026). Four of ten participants (40%) achieved MAE ≤ 2.0 bpm (Participants 1–4; mean MAE 1.0 bpm), consistent with the simulation resting condition (MAE = 0.46 bpm). The remaining six participants showed errors of 6–17 bpm attributable to identifiable signal characteristics described below.

Table 2 error-source classification criteria: None—|BRest − BRref| ≤ 3 bpm; Nonresp. HF osc.—dominant spectral peak at LF–HF boundary (0.15–0.17 Hz) with SNR ≥ 3.0 and estimated frequency diverging > 5 bpm from the independent respiratory reference; Subharmonic—BRref/BRest ≈ 2.0 (ratio 1.8–2.2); Competing HF peak—SNR 2–5 with frequency shift > 5 bpm and no harmonic ratio pattern; 2nd Harmonic—BRest/BRref ≈ 2.0 (ratio 1.8–2.2). Classification was assigned by post hoc spectral inspection of each participant’s Welch PSD; prospective automated detection using these quantitative thresholds is proposed as future work.

Table 2 presents per-subject validation results using real recordings, comparing the reference respiratory rate (BR_ref_) obtained from thermistor FFT with the estimated rate (BR_est_) derived from the HRV HF peak.

Absolute error (Err) and HF-SNR are reported for each participant, along with identified confounding factors that explain estimation failures. Participants marked with an asterisk (*) have HF-SNR values below the quality-gate threshold (SNR < 2.0), indicating that their estimates would be rejected according to Equation (5).

Three distinct failure modes were observed:

1. Non-respiratory HF oscillations

Participants 6 and 9 exhibited dominant HRV peaks near 0.15 Hz (LF–HF boundary), unrelated to respiration, despite high SNR values. Participant 9 demonstrates that high SNR does not guarantee respiratory origin when competing oscillations are present.

2. Harmonic confusion

Participant 10 breathed at 12 bpm (0.20 Hz), but the HRV peak appeared at 24 bpm (0.40 Hz), corresponding to the second harmonic of a non-sinusoidal RSA waveform.

3. Sub-harmonic or competing HF peaks

Participants 5 and 7 showed HRV peaks at sub-harmonic or competing frequencies, indicating multi-modal HF spectra.

Participant 8 marginally passed the SNR quality gate (SNR = 2.14, just above the rejection threshold of 2.0), yet incurred an error of 6 bpm—a boundary-case failure in which the threshold provided insufficient protection because RSA amplitude was genuinely weak rather than inflated by a non-respiratory oscillation. This boundary class is distinct from the high-SNR failure of Participant 9 (SNR = 7.88, error = 17 bpm) and motivates a graduated response strategy: mandatory supplementary quality checks (peak-stability and harmonic ratio tests) applied specifically in the marginal range SNR = 2.0–3.0, rather than a binary accept/reject gate at a single threshold.

Overall accuracy was MAE = 6.80 bpm across all 10 participants, but MAE = 1.00 bpm for the four participants without identifiable confounders.

The discrepancy between simulation accuracy (MAE = 0.46 bpm under normal rest; 0.46–3.45 bpm across five simulated conditions) and real-world performance (mean MAE = 6.8 bpm; range 0–17 bpm; six of 10 participants exceeding a 3 bpm clinical threshold) is quantitatively large: mean real-world error exceeded the worst simulated condition by approximately two-fold. Critically, simulation predicted no failures at SNR ≥ 2.0; in the real cohort, six of 10 participants incurred clinically relevant errors (6–17 bpm) despite passing the quality gate. This gap reflects confounders not captured in the simulation framework, including non-respiratory HF oscillations, harmonic confusion, and breathing waveform non-stationarity—effects amplified in older adult participants with reduced vagal tone.

## 3. Discussion

### 3.1. Physiological Validity of HF Peak = Respiratory Frequency

RSA arises from vagally mediated modulation of sinus node firing by pulmonary stretch receptors and central respiratory drive. The resulting RR oscillation is phase-locked to the breathing cycle, with heart rate accelerating during inhalation and decelerating during exhalation [4]. The HF spectral peak frequency is therefore a direct readout of respiratory frequency, provided RSA coupling is present. This coupling is robust across the physiological breathing range (0.15–0.40 Hz = 9–24 bpm) in young-to-middle-aged healthy adults. In older adult participants, RSA coupling may be diminished due to age-related vagal withdrawal, but our simulation shows that reduced RSA amplitude alone does not impair frequency estimation—only when it combines with other factors to reduce SNR below ~1.5.

### 3.2. Respiratory Irregularity as the Dominant Simulation Error Source

In our simulation, respiratory irregularity was the dominant error source (MAE 3.5–4.0 bpm), consistent with the spectral mechanism: breathing frequency drift disperses HF power and reduces peak prominence. This finding is confirmed by published data: Charlton et al. [5] noted that frequency-estimation accuracy degrades sharply when respiratory rate varies within the analysis window.

### 3.3. Real-Data Failure Modes in Elderly Participants

The real-data validation revealed three failure modes. First, non-respiratory HF oscillations at the LF–HF boundary (~0.15 Hz) dominated Participants 6 and 9 (errors 16–17 bpm), confirming that high SNR is necessary but not sufficient: Participant 9 achieved SNR = 7.88 yet incurred 17 bpm error. Second, spectral confusion—sub-harmonic detection (Participant 5), competing HF peaks (Participant 7), and 2nd-harmonic aliasing (Participant 10)—produced errors of 6–12 bpm, a failure class the univariate SNR criterion cannot detect. Third, Participant 8 (SNR = 2.14, error = 6 bpm) illustrates the quality-gate boundary: a value just above the SNR = 2.0 threshold coincided with weak RSA, underscoring that the threshold is conservative, not guaranteed. These findings motivate peak-stability checking and harmonic ratio testing as supplementary quality criteria.

### 3.4. Sleep as a Favorable—But Stage-Dependent—Application Context

HRV-HF respiratory estimation is best suited to NREM sleep (N1–N3), where autonomous brainstem regulation produces stable breathing at 12–16 breaths/min with intact RSA—conditions within the algorithm’s optimal operating range. REM sleep introduces irregular breathing (σ_f up to 0.06–0.08 Hz), sympathetic bursts, and reduced RSA amplitude that collectively degrade SNR. In practice, NREM estimates with SNR ≥ 2.0 can be used with confidence; REM estimates should be flagged or supplemented with respiratory effort sensors until multi-modal validation is available. These stage-specific recommendations are based on physiological reasoning and simulation-based extrapolation, not on direct experimental observation during sleep; they should be treated as working hypotheses pending polysomnography-concurrent empirical validation.

### 3.5. Beyond SNR: Towards Robust Quality Criteria

The demonstration that Participant 9 had SNR = 7.88 yet MAE = 17 bpm highlights a fundamental structural limitation of the current quality metric: it cannot distinguish respiratory RSA from non-respiratory HF oscillations. Quantitatively, Participant 9’s HRV spectrum exhibited a dominant peak near 0.15 Hz (≈9 bpm)—at the LF–HF boundary—rather than at the true respiratory frequency of approximately 0.30 Hz (≈18 bpm). The peak-to-mean-band-power ratio of 7.88 reflected exceptional spectral sharpness at 0.15 Hz, consistent with a narrow-band baroreflex or postural low-frequency oscillation rather than RSA-driven respiration. Because the SNR formula (Equation (5)) quantifies only the sharpness of the dominant spectral peak—irrespective of its physiological origin—any sufficiently narrow-band non-respiratory oscillation within the HF band will produce a high SNR score even when the true respiratory peak is subthreshold. This exposes a categorical gap: the current metric screens for spectral quality but cannot verify respiratory identity. Candidate additional quality criteria to close this gap include:

1. Cross-spectral coherence between HF-peak frequency and a secondary respiratory signal (e.g., accelerometry-based respiration or pulse transit time) at the estimated frequency.
2. Intra-window peak stability: the standard deviation of HF peak frequency across sub-windows (reject if SD > 1.5 bpm/min).
3. Harmonic ratio test: verify that the estimated frequency is not the second harmonic of an LF oscillation (flag if power at f_est/2 exceeds power at f_est).
4. Machine-learning-based SNR proxies trained on labeled real-data segments could provide more robust quality estimates than the current univariate formula.
5. Of the four supplementary criteria above, intra-window peak stability (Criterion 2) and the harmonic ratio test (Criterion 3) are recommended as the highest-priority additions for resource-constrained wearable implementations: both are computed from the same Welch PSD generated in the main pipeline (Section 3.6, Stage 3) without additional hardware. Specifically, a peak-stability SD threshold of ≤1.5 bpm/min and a harmonic-power criterion (power at f_est/2 < power at f_est) should be adopted alongside SNR ≥ 2.0 as the minimum viable quality suite. Cross-spectral coherence with a secondary respiratory channel (Criterion 1) provides the strongest mechanistic rejection of non-respiratory HF oscillations but requires a co-located accelerometer or pulse oximeter—appropriate for polysomnography-grade validation rather than minimal-sensor wearables. Machine-learning SNR proxies (Criterion 4) offer the highest potential accuracy ceiling but require labeled real-world training data for this population, which are not yet publicly available.

### 3.6. Wearable Implementation: Five-Stage Pipeline

Based on simulation and real-data findings, we recommend the following five-stage pipeline for wearable implementation:

1. RR/PPI preprocessing: cubic spline interpolation of outlier intervals (|RR_n − median| > 25%).
2. Accelerometry-gated exclusion: reject windows with RMS acceleration > 0.05 g.
3. Adaptive window length: 60 s for stable breathing; 30 s for potentially drifting breathing.
4. SNR quality gate (Equation (5)): withhold estimate when SNR < 2.0. Supplement with peak-stability and harmonic ratio tests in older adult populations.
5. Adaptive HF band: extend lower bound to 0.10 Hz [10,11,12,13,14,15] for older adult users and NREM sleep; extend upper bound to 0.45 Hz for fast-breathing participants. Flag estimates within 2 bpm of band boundaries as potentially unreliable.

### 3.7. Limitations

Several limitations of the present study warrant consideration. First, while the physiologically calibrated simulation provided a controlled environment for error characterization, it modeled the RSA coupling as a pure sinusoidal wave. This approach inherently simplifies the complex waveform asymmetries and non-stationarity characteristic of real-world respiratory signals. Our real-data empirical findings confirmed that harmonic confusion—a direct consequence of non-sinusoidal RSA morphology—represents a critical failure mode in older adult individuals that was not fully captured by the simulation framework. Second, the real-data validation cohort was relatively small (N = 10) and restricted to healthy community-dwelling older adult participants. Pathological cardiac and respiratory conditions, such as atrial fibrillation, premature ventricular contractions, obstructive sleep apnea, and chronic heart failure, substantially alter the underlying HRV spectral dynamics. Consequently, these conditions require condition-specific validation. To achieve larger-scale and statistically robust validation, public datasets containing concurrent cardiac and respiratory references—such as the BIDMC PPG and Respiration Dataset, the MIMIC-III Waveform Database, and the Fantasia Database—should be utilized in future investigations. Third, the proposed HF-SNR metric (the ratio of peak power to mean band power) effectively quantifies spectral peak sharpness but lacks the mechanistic capacity to distinguish true vagally mediated RSA from non-respiratory HF oscillations. This physiological limitation was exemplified by a false-confidence failure mode in Participant 9, who exhibited a high SNR of 7.88 despite a severe estimation error (MAE = 17 bpm). To mitigate this vulnerability, more robust multimodal quality criteria must be integrated, as discussed in Section 3.5. Fourth, the derived empirical power-law formula accounted for 71% of the total error variance (R^2^ = 0.71). The remaining 29% of unexplained variance reflects complex, non-linear interactions between respiratory irregularity, baseline noise, and ectopic artifact rates. Although the univariate formula remains highly recommended for practical, low-computational quality gating in wearable devices, a multivariate model could further improve error-prediction precision. Finally, the designation of NREM sleep as an optimal context for this estimation method was inferred through mechanistic reasoning and simulation-based extrapolation rather than direct observation. Empirical validation using polysomnography (PSG)-scored sleep-stage data remains necessary, as the real-data validation in this study was conducted during awake, seated rest rather than actual sleep. Furthermore, pathological irregular breathing patterns (e.g., Cheyne–Stokes respiration or central apneas) and transient respiratory events (e.g., sighs) were modeled solely as stochastic frequency jitter. The distinct spectral signatures of these periodic and episodic breathing disorders require dedicated mathematical modeling in future clinical extensions.

Additional limitations of this pilot study require acknowledgement. The sample size of 10 older adult participants limits statistical power and generalizability; future studies with larger cohorts incorporating power analysis are needed to validate the proposed error model across diverse populations and sleep conditions. The five-stage adaptive pipeline components—cubic-spline preprocessing, accelerometry gating, adaptive window length, SNR quality gate, and adaptive HF-band extension—have not been independently validated against held-out datasets; the incremental accuracy benefit of each stage remains to be quantified. The HF-band extension rule (lower bound 0.10 Hz for older adults; upper bound 0.45 Hz for fast breathers) is supported by published physiological references [16,17,18,19,20,21] but was not tested against the present cohort; the risk of admitting LF-boundary noise at the lower extension, and the gain from the upper extension, remain unquantified. The regression diagnostic was applied as follows: Cook’s distance (Di > 4/n) identified influential observations and leverage points; multicollinearity was assessed using Variance Inflation Factors (VIF < 5 threshold). Confidence intervals for the empirical coefficients C and α are provided in Section 4.5. Finally, the claim that NREM sleep is an optimal application context rests on physiological reasoning and simulation-based extrapolation, not direct experimental evidence; polysomnography-concurrent recordings validating performance across sleep stages are required before clinical recommendations can be made. Future work should also include polysomnography-concurrent recordings and participants with pathological breathing patterns to extend generalizability.

## 4. Materials and Methods

### 4.1. ECG Dataset for Pipeline Validation

ECG processing pipeline validation used the PhysioNet ECG-ID Database (Electrocardiogram Identification Database; Lugovaya, 2005 [9]; PhysioNet platform: Goldberger et al., 2000 [22]; DOI: 10.13026/C2XW26). As required by PhysioNet citation guidelines [22], both the dataset and the platform are cited. The database contains 310 single-lead ECG recordings (Lead I) from 90 healthy volunteers (44 male, 46 female; age 13–75 years), each 20 s in duration, sampled at 500 Hz with 12-bit resolution (range ± 10 mV). Because individual recordings are 20 s in duration—shorter than the 30 s analysis window required for HF frequency resolution—and because the database contains no simultaneous respiratory reference signal, the ECG-ID Database was used solely to validate the RR-interval extraction and Welch PSD pipeline on real cardiac signals, not for error characterization. Error characterization, which requires controlled respiratory ground truth and sustained recordings, was performed using the simulation framework described in Section 4.4, following established methodology [5,6].

**Figure 2:**
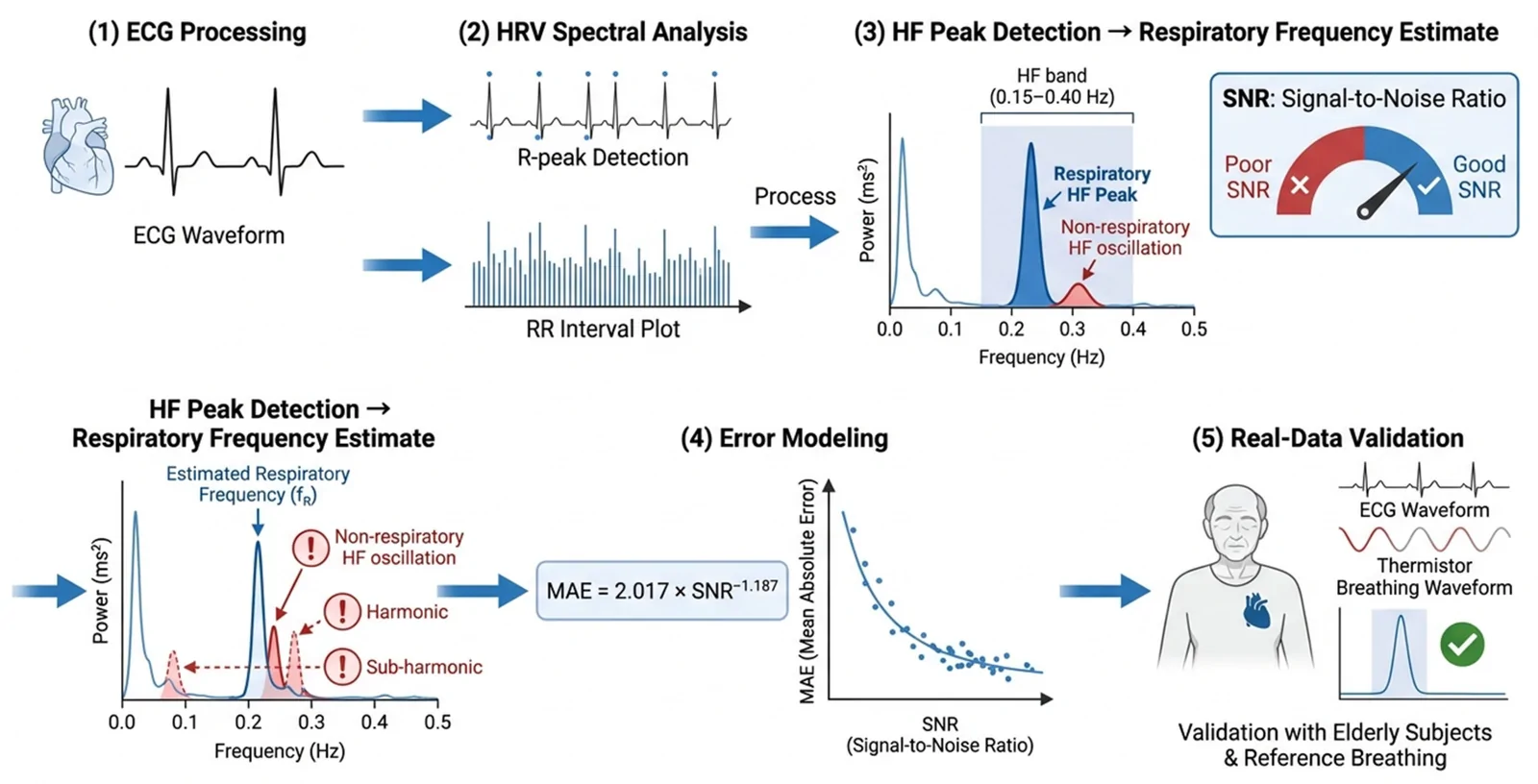
HRV-based respiratory frequency estimation workflow. ECG signals are converted to RR intervals and analyzed by Welch PSD. The dominant HF spectral peak (0.15–0.40 Hz) is extracted and converted to breathing rate (BR = 60 × f_peak Hz). HF-SNR (Equation (5)) gates estimate quality; outputs with SNR < 2.0 are withheld. The empirical error model (Equation (6)) provides MAE bounds as a function of SNR. Validation cohort: 10 older adult participants (mean age 71.3 years).

Figure 2 illustrates the overall processing protocol used in this study, including RR-interval extraction, artifact handling, and Welch PSD computation for HF peak detection. This protocol was applied consistently across both simulated and real ECG recordings.

### 4.2. Real-Data Validation: Participants and Protocol

For real-data validation with simultaneous respiratory ground truth, we recruited 10 community-dwelling older adult volunteers (five male, five female; age range 61–82 years; mean 71.3 ± 6.1 years) who participated in laboratory physiological measurements on 1–2 March 2026. Exclusion criteria were known cardiac arrhythmia, active pulmonary disease, and use of medications known to suppress HRV. All participants provided written informed consent. The study was conducted in accordance with the Declaration of Helsinki. This study was approved by the Ethics Review Committee of the Graduate School of Engineering, Mie University (Approval No. 155; approved 4 February 2026). Multi-channel physiological signals were recorded using bio-signal amplifier (Biotope Mini, (East Medic Inc, Japan) at 1000 Hz sampling rate. Channel 1 (Ch1) recorded single-lead ECG; Channel 6 (Ch6) recorded nasal/oral thermistor airflow signal as the respiratory reference. Ch6 was confirmed as the respiratory channel by spectral power ratio analysis (0.1–0.5 Hz respiratory band power/total power = 0.88–0.93 across participants, compared to 0.001–0.058 for the ECG channel). Participants rested in a seated position for a minimum of 5 min before recording. A 120 s analysis segment was extracted starting at t = 30 s to avoid initial transients.

### 4.3. RR Interval Extraction

R-peaks were identified using a derivative-threshold detector with 400 ms baseline removal (moving-average filter) and adaptive 97th-percentile threshold. A 400 ms refractory period prevented double detection. Ectopic beats and outlier RR values (|RR_n_ − median (RR)| > 25% × median (RR)) were excluded before interpolation. The RR interval series was interpolated to a uniform 4 Hz time grid using linear interpolation. Power spectral density was estimated using Welch’s method: window length 30 s (simulation) or 60 s (real-data validation), Hann taper, 50% overlap, consistent with Task Force guidelines [4]. The HF band (0.15–0.40 Hz; extended to 0.45 Hz for participants with breathing rates above 23 bpm, i.e., above the standard upper HF boundary) was searched for the dominant spectral peak:

(3) $$f_{resp}[Hz]=argmax\_\{f\;in\;HF\}PSD(f)$$

The HF signal-to-noise ratio (HF-SNR) was computed as the ratio of peak power to mean band power—a spectral Q-factor measure that quantifies respiratory peak sharpness relative to background HF energy [5,23]. This index is computable without a respiratory reference signal and is therefore suitable for real-time wearable quality assessment:

(4) $$SNR=P_{peak}/mean(P_{HF})$$

### 4.4. Simulation Framework for Error Characterization

To isolate the individual contribution of each signal quality factor (RSA amplitude, respiratory irregularity, noise level, and artifact rate)—which cannot be independently manipulated in real recordings—a physiologically calibrated simulation was used as complementary analysis. This approach follows established methodology for evaluating HRV-based respiratory estimators in the absence of a synchronous respiratory reference [5,6,23]. The simulation is not intended to substitute for real-data validation but to provide controlled error characterization across systematically varied conditions.

Beat-level RR intervals were generated as:

(5) $${RR}_{n\;}=\;{RR}_{mean}\;+\;A_{RSA}\cdot \;sin(2\pi f_{resp}t_{n})\;+\;\varepsilon_{n}\;$$

The following are defined here:

*RR*_*n*_: n-th RR interval

*RR*_mean_: mean RR interval

*A*_RSA_: RSA (Respiratory Sinus Arrhythmia) amplitude

*f*_resp_: respiratory frequency

*t*_*n*_: time corresponding to the n-th RR interval

*ϵ*_*n*_: noise/residual

Where RR_mean = 60/HR_mean, A_RSA is the RSA coupling amplitude, f_resp is the true respiratory frequency, t_n is cumulative beat time, and epsilon_n ~ N(0, sigma_noise). The sinusoidal RSA model represents the dominant linear coupling mechanism; real RSA is non-sinusoidal and modulated by respiratory waveform asymmetry. Five conditions (N = 30 participants, 180 s duration) were evaluated and are shown in Table 4.

RSA (Respiratory Sinus Arrhythmia) is a phenomenon in which the heartbeat interval (RR interval) fluctuates cyclically in sync with respiration. The heart rate increases (and the RR interval shortens) during inspiration (inhaling), while it decreases (and the RR interval lengthens) during expiration (exhaling). As this physiological phenomenon reflects the activity of the autonomic nervous system (particularly the vagus nerve), it serves as a crucial indicator in heart-rate variability (HRV) research.

### 4.5. Reference Respiratory Rate from Thermistor

For the real-data cohort, the thermistor respiratory reference frequency was determined as the dominant spectral peak (0.10–0.55 Hz) of the linearly detrended thermistor signal using a 60 s Hann-windowed FFT. For participants where the FFT dominant peak exceeded the zero-crossing-based count by more than 5 bpm, a note of respiratory waveform non-stationarity was recorded (see Section 2.4).

### 4.6. Empirical Error Formula Derivation

To establish a predictive model for estimation accuracy in real-time wearable applications where a respiratory reference signal is absent, we formulated an empirical error prediction model based on the spectral properties of the HRV signal. A log-linear regression analysis was performed on a calibration dataset comprising 600 synthetic RR-interval recordings (180 s duration each). These synthetic signals were generated by systematically permuting the generative parameters: the RSA amplitude (A_RSA_) was varied from 0.01 to 0.08 s in steps of 0.01 s, and the additive white noise standard deviation (σ_noise_) was varied from 0.005 to 0.025 s in steps of 0.005 s. For each generated recording, the true mean absolute error (MAE) and the corresponding high-frequency signal-to-noise ratio (HF-SNR) were extracted using the pipelines described in Section 4.4. The underlying relationship between the estimation error and the spectral signal quality was modeled using a power-law function:

(6) $$MAE=C\cdot {SNR}^{\alpha }$$

where MAE is the mean absolute error in breaths per minute (bpm), SNR is the non-dimensional HF-SNR defined in Equation (3), C is the scaling coefficient representing the baseline error under unity SNR, and α is the power-law exponent dictating the sensitivity of the error to changes in signal quality. To estimate the parameters C and α using ordinary least squares (OLS) regression, a natural logarithmic transformation was applied to both sides of the equation, yielding the following linear framework:

(7) ln(MAE)=ln(C)+αln(SNR)+e

where e represents the homoscedastic residual error term. Influential observations and leverage points were identified using Cook’s distance (threshold Di > 4/n, where n is the sample size); multicollinearity was assessed using Variance Inflation Factors (VIF; threshold VIF < 5). The goodness-of-fit was evaluated using the coefficient of determination (R^2^), and the 95% confidence intervals (CIs) for both C and α were calculated to confirm the statistical reliability of the derived empirical formula.

### 4.7. Evaluation Metrics and Quality Gating

The performance of the HRV-based respiratory frequency estimator was evaluated against the definitive ground truth (the calibrated simulated breathing rate for the synthetic dataset, and the thermistor-derived respiratory rate for the real-data validation cohort). For each analysis window, the estimation error (ΔBR) was defined as:

(8) $${\Delta BR}_{i}={BR}_{est,i}-{BR}_{ref,i}$$

where BR_est,i_ is the estimated breathing rate derived from the HF spectral peak (60 × f_resp_) and BR_ref,i_ is the corresponding reference breathing rate in bpm. The overall accuracy across the cohort or experimental conditions was quantified using three complementary statistical metrics: Mean Absolute Error (MAE), Root Mean Square Error (RMSE), and the 95th-percentile Absolute Error (P_95_). These metrics are formalized as follows:

(9) $$MAE=\frac{1}{N}\sum_{ᵢ₌₁}^{ⁿ}\left|\Delta BRᵢ\right|$$

(10) $$RMSE=\sqrt{\frac{1}{N}\sum_{ᵢ₌₁}^{ⁿ}{\left(\Delta BRᵢ\right)}^{2}}$$

(11) P₉₅=P₉₅({|ΔBR₁|,|ΔBR₂|,…,|ΔBRₙ|})

where N denotes the total number of valid analysis windows. MAE reflects the average expected error magnitude, RMSE penalizes larger tracking errors or harmonic confusion outliers more heavily, and P_95_ serves as an indicator of the worst-case error boundary, which is a critical safety and reliability parameter for clinical sleep monitoring.

Furthermore, to mimic the adaptive data-rejection algorithms embedded in commercial wearable sensors, an autonomous SNR-based quality-gating criterion was established. Using the empirical error formula derived in Section 4.6, a threshold was set to exclude highly degraded or uncoupled segments. Specifically, any analysis window with an estimated HF-SNR that predicted an MAE > 1.0 bpm (which mathematically corresponds to an SNR threshold below 2.0) was flagged as “low quality” and rejected from the final per-subject accuracy statistics. The data retention rate (the ratio of accepted windows to total recorded windows) was calculated for each participant to evaluate the trade-off between strict quality gating and continuous monitoring availability, particularly in the older adult population where transient non-respiratory HF components are highly prevalent.

## 5. Conclusions

Respiratory frequency can be estimated from the HF component of HRV with MAE < 1 bpm under resting and simulated sleep conditions, based on the principle that RSA couples breathing frequency directly to the RR interval spectrum. The dominant error source in simulation is respiratory irregularity (MAE 3.5–4.0 bpm), not RSA amplitude reduction. An empirical error formula (MAE = 2.017 × SNR^(−1.187)) enables real-time quality gating.

Real-data validation in 10 older adult participants revealed three additional failure modes absent from the simulation: non-respiratory HF oscillations with high spectral purity, harmonic confusion from non-sinusoidal RSA waveforms, and breathing rate instability. Four of ten participants achieved MAE ≤ 2.0 bpm; the remaining six showed the identified failure modes. The SNR quality gate correctly flagged one low-SNR participant but failed for the non-respiratory oscillation case (SNR = 7.88, MAE = 17 bpm), motivating the development of more robust quality criteria including cross-spectral coherence, intra-window peak stability, and harmonic ratio tests. NREM sleep represents the most favorable wearable deployment context: stable autonomous breathing, enhanced parasympathetic tone, and minimal body motion collectively suppress the failure modes identified in the real-data cohort. These findings are directly relevant to circadian rhythm assessment, sleep staging, and fatigue monitoring.

## Figures and Tables

**Table 1:** Respiratory-rate estimation accuracy across five simulation conditions.

Condition	MAE (bpm)	RMSE (bpm)	P95 (bpm)	HF-SNR
Normal rest	0.46	0.54	0.89	4.8
Mild irregular breathing	3.95	4.55	6.78	1.6
RSA weak	0.51	0.60	0.97	4.0
Severe irregular breathing	3.51	4.11	6.30	1.5
High noise (8% artifact)	1.72	2.47	5.52	1.8

N = 30 per condition, 180 s recordings.

**Table 2:** Per-subject real-data validation results.

Sub	Gender	Age	BR_ref_	BR_est_	Err (bpm)	SNR	Error Source
1	M	74	23.0	23.0	0.00	3.71	None
2	M	61	22.0	20.0	2.00	4.28	None
3	M	82	16.0	14.0	2.00	5.45	None
4	F	66	20.0	20.0	0.00	2.89	None
5	F	75	16.0	10.0	6.00	4.09	Sub-harmonic
6	F	72	25.0	9.0	16.00	3.07	Non-resp. HF oscillation
7	F	64	14.0	21.0	7.00	3.39	Competing HF peak
8 *	F	71	18.0	12.0	6.00	2.14	Weak RSA (below quality gate)
9	M	73	26.0	9.0	17.00	7.88	Non-resp. HF oscillation (high SNR)
10	M	75	12.0	24.0	12.00	2.28	2nd harmonic detection

Participants marked with an asterisk (*) have HF-SNR values below the quality-gate threshold (SNR < 2.0).

**Table 3:** Predicted MAE as a function of HF-SNR.

HF-SNR	Predicted MAE (bpm)	Quality Interpretation
1.2	1.62	Marginal
1.5	1.25	Low quality
2.0	0.89	Moderate—quality gate threshold
3.0	0.55	Good
5.0	0.30	High
8.0	0.17	Excellent

**Table 4:** Simulation conditions.

Condition	A_RSA_ (s)	Noise SD (s)	Irreg.	Artifact
Normal rest	0.05	0.008	No	0%
Mild irregular breathing	0.04	0.012	Yes (±0.02 Hz)	0%
RSA weak (stress/older adults)	0.02	0.010	No	0%
Severe irregular breathing	0.04	0.015	Yes (±0.05 Hz)	0%
High noise (8% artifact)	0.04	0.010	No	8%

A_RSA_: RSA amplitude; Irreg.: frequency jitter; Artifact: artifact rate.

## Data Availability

The data supporting the findings of this study are not publicly available due to privacy and ethical restrictions. The physiological recordings (ECG-derived RR intervals and respiratory signals) contain identifiable human biometric information, and public release is not permitted under the conditions of the ethics approval issued by the Ethics Review Committee of the Graduate School of Engineering, Mie University (Approval No. 155). ECG pipeline validation data are publicly available: PhysioNet ECG-ID Database (Electrocardiogram Identification Database), DOI: 10.13026/C2XW26, https://physionet.org/content/ecgiddb/ (accessed on 24 August 2026). Real-data validation data (10-subject Biotope Mini recordings) are available from the corresponding author upon reasonable request, subject to participant and ethics constraints.

## Abbreviations

The following abbreviations are used in this manuscript:

ARSA/$A_{\mathrm{RSA}}$	Respiratory Sinus Arrhythmia Amplitude
BR	Breathing Rate
CI	Confidence Interval
ECG	Electrocardiogram
FFT	Fast Fourier Transform
HF	High-Frequency
HF-SNR	High-Frequency Signal-to-Noise Ratio
HRV	Heart-Rate Variability
MAE	Mean Absolute Error
NREM	Non-Rapid Eye Movement
OLS	Ordinary Least Squares
PSD	Power Spectral Density
RMSE	Root Mean Square Error
RR	R-wave to R-wave (Interval)
RSA	Respiratory Sinus Arrhythmia
SNR	Signal-to-Noise Ratio
